# Nickel Toxicity Interferes with NO_3_^−^/NH_4_^+^ Uptake and Nitrogen Metabolic Enzyme Activity in Rice (*Oryza sativa* L.)

**DOI:** 10.3390/plants11111401

**Published:** 2022-05-25

**Authors:** Muhammad Rizwan, Kamal Usman, Mohammed Alsafran, Hareb Al Jabri, Tayyaba Samreen, Muhammad Hamzah Saleem, Shuxin Tu

**Affiliations:** 1 Office of Academic Research, Office of VP for Research & Graduate Studies, Qatar University, Doha 2713, Qatar; m.rizwan@qu.edu.qa (M.R.); saleemhamza312@webmail.hzau.edu.cn (M.H.S.); 2 College of Resources and Environment, Huazhong Agricultural University, Wuhan 430070, China; 3 Agricultural Research Station (ARS), Office of VP for Research and Graduate Studies, Qatar University, Doha 2713, Qatar; kusman@qu.edu.qa; 4 Central Laboratories Unit (CLU), Office of VP for Research and Graduate Studies, Qatar University, Doha 2713, Qatar; 5 Center for Sustainable Development, College of Arts and Sciences, Qatar University, Doha 2713, Qatar; h.aljabri@qu.edu.qa; 6 Department of Biological and Environmental Sciences, College of Arts and Sciences, Qatar University, Doha 2713, Qatar; 7 Institute of Soil and Environmental Sciences, University of Agriculture, Faisalabad 38040, Pakistan; tybasamreen@gmail.com

**Keywords:** rice, metabolism, nitrogen, nickel, stress

## Abstract

The excessive use of nickel (Ni) in manufacturing and various industries has made Ni a serious pollutant in the past few decades. As a micronutrient, Ni is crucial for plant growth at low concentrations, but at higher concentrations, it can hamper growth. We evaluated the effects of Ni concentrations on nitrate (NO_3_^−^) and ammonium (NH_4_^+^) concentrations, and nitrogen metabolism enzyme activity in rice seedlings grown in hydroponic systems, using different Ni concentrations. A Ni concentration of 200 μM significantly decreased the NO_3_^−^ concentration in rice leaves, as well as the activities of nitrate reductase (NR), nitrite reductase (NiR), glutamine synthetase (GS), and glutamate synthetase (GOGAT), respectively, when compared to the control. By contrast, the NH_4_^+^ concentration and glutamate dehydrogenase (GDH) activity both increased markedly by 48% and 46%, respectively, compared with the control. Furthermore, the activity of most active aminotransferases, including glutamic pyruvic transaminase (GPT) and glutamic oxaloacetic transaminase (GOT), was inhibited by 48% and 36%, respectively, in comparison with the control. The results indicate that Ni toxicity causes the enzymes involved in N assimilation to desynchronize, ultimately negatively impacting the overall plant growth.

## 1. Introduction

Heavy metals mainly accumulate in agricultural soils via natural and anthropogenic sources, such as weathering, mining, waste disposal, and the excessive use of pesticides and fertilizer [1,2]. Soil polluted with heavy metals reduces agricultural land quality, and crop yield and quality [3,4], as well as induces deleterious effects on the soil biota [5]. Plants exposed to high Ni concentrations are affected by a variety of toxic effects, including seed germination inhibition, stunted growth [3], leaf chlorosis induction, wilting, necrosis, enhanced lipid peroxidation [6], as well as reduced yields. Previous studies reported that Ni toxicity is linked to plants’ oxidative damage [7,8]. In support of this fact, studies found that Ni could interfere indirectly with the antioxidant system responses [9]. Similarly, Ni toxicity resulted in decreased plant height, rice yield, and nitrogen, phosphorus, and potassium concentrations in rice crops [10], and reduced chlorophyll contents and biomass production in *Eruca sativa* plants [2].

Ni, a component of the plant, soil, and aquatic environment, is an essential micronutrient in low concentrations, which is required for plants, such as cotton (*Gossypium hirsutum* L.), wheat (*Triticum aestivum* L.), potato (*Solanum tuberosum* L.), tomato (*Solanum Lycopersicon* L.), and other plant species, to complete their growth cycles [11]. Ni is a vital component of urease and is also a constituent of several metalloenzymes, such as Ni-Fe hydrogenase, methyl-coenzyme M reductase, acetyl coenzyme-A synthase, RNase-A, and superoxide dismutase [12]. Ni toxicity decreases the nitrogen concentration, inhibits nitrate uptake, and negatively influences nitrogen assimilation-related enzymes. Although attempts have been made to explore Ni toxicity mechanisms in plants, the impact of Ni on primary plant metabolism remains poorly understood.

Nitrogen (N) is considered to be an essential macronutrient for plant growth [13,14], and also an important component of many organic molecules, such as proteins and amino acids, which regulate processes including N assimilation, antioxidant defense systems, photosynthesis, carbohydrate metabolism, and the entire cellular cycle [15]. Plants usually take up N through nitrate (NO_3_^−^) and ammonium (NH_4_^+^) ions from the soil. Firstly, the reduction of NO_3_^−^ into nitrite (NO_2_^−^) is catalyzed by cytosolic nitrate reductase (NR), then nitrite reductase (NiR) located in the chloroplasts converts NO_2_^−^ into NH_4_^+^. Secondly, glutamine synthetase (GS) and glutamate synthase (GOGAT) collaborate to incorporate NH_4_^+^ into organic compounds [16]. The glutamate dehydrogenase (GDH) assimilates NH_4_^+^ ions into organic compounds, in addition to the GS/GOGAT cycle. GDH plays a limited or no role in the primary assimilation of NH_4_^+^ in plants, because of its affinity with NH_4_^+^, in relation to GS [17]. However, GDH plays a pivotal role in stressful environments when the GS/GOGAT system is inhibited. GDH activity induction can mitigate the accumulation of toxic quantities of NH_4_^+^ and provide the glutamate needed for several protective biomolecules [18]. Finally, glutamate can be transaminated into other amino acids by enzymes known as glutamic oxaloacetic transaminase (GOT) and glutamic pyruvic transaminase (GPT) [19,20].

A few studies examined Ni tolerance and the causes of rice toxicity prior to this study. Nevertheless, little is known about how Ni affects rice growth and N metabolism during seedling development. Therefore, it is necessary to carry out in-depth analyses to understand the N assimilation capacity of the rice crop under Ni stress, to counter the problems associated with Ni toxicity. In this study, we investigated the effects of Ni on growth, the content of photosynthesis pigments, and the enzyme activities of N metabolism in rice seedlings grown under a gradient of Ni concentrations in a controlled hydroponics system. The study will assist us in understanding how plants manage their nitrogen metabolism in stressed conditions.

## 2. Results

### 2.1. Ni-Induced Visible Toxicity Symptoms and Ni Contents in Rice Plants

**Figure 1:**
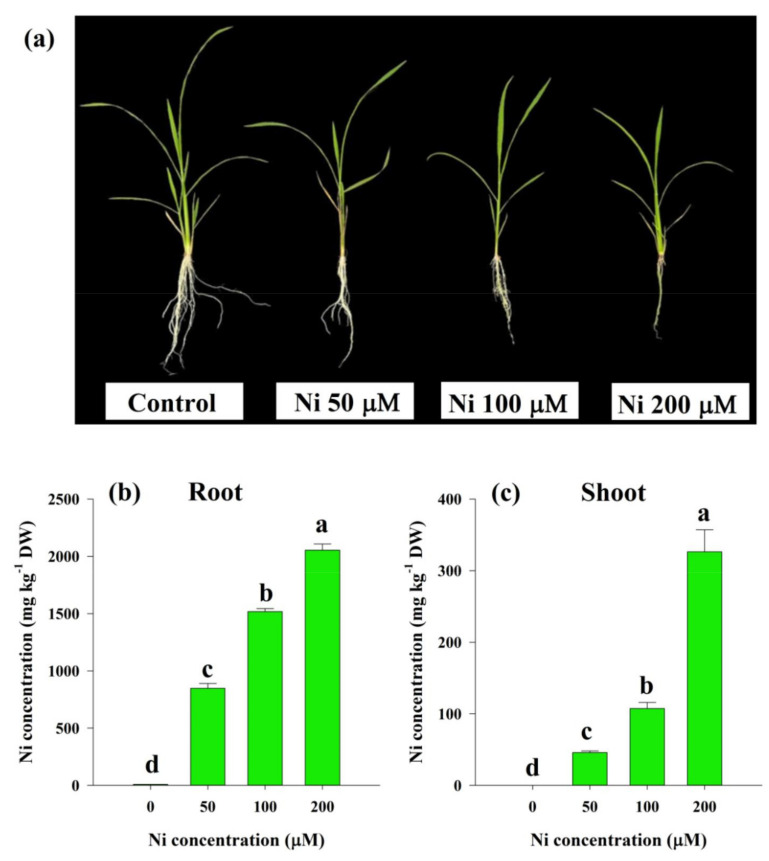
Symptoms of nickel (Ni) toxicity (**a**), Ni concentrations in the root (**b**), and shoot (**c**) of rice seedlings subjected to 0, 50, 100 and 200 μM Ni for a period of 14 days. Bars represent means ± standard deviations (SDs) of three independent replications (*n* = 3). Means followed by the same letter did not significantly differ among the treatments at *p* < 0.05, according to Fisher’s least significant difference test.

We measured the root and shoot Ni concentration in response to Ni stress, in order to determine whether the Ni concentration correlates with rice plant toxicity. Higher Ni concentrations caused more symptoms of Ni toxicity and a higher accumulation of Ni in the roots and shoots, as compared with the control plants (Figure 1). Ni toxicity was observed on the rice seedling leaves, which showed symptoms of chlorosis, as well as necrosis, and this was most apparent on the new leaves. In relation to higher concentrations of Ni in the roots and shoots of Ni-stressed rice plants, we found that excessive Ni severely hampered rice growth performance by reducing plant height, as well as showing shorter and less hairy roots than the control plants (Figure 1a). The Ni concentration was increased by 109-, 195-, and 264-fold in the roots, and 687-, 1610-, and 4894-fold in the shoots at 50, 100, and 200 μM Ni, respectively, in relation to the control (Figure 1b,c).

### 2.2. Impact of Ni Stress on Plant Growth, Biomass, and Photosynthetic Pigments

**Figure 2:**
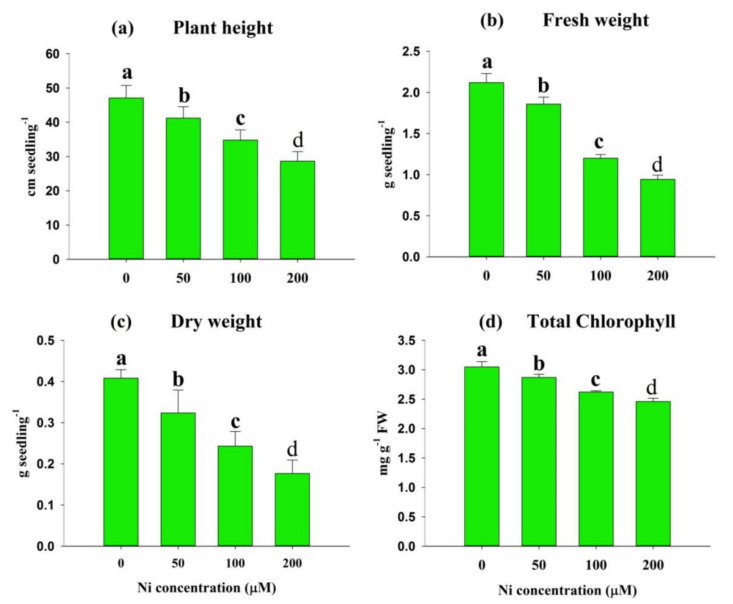
Effects of nickel (Ni) on plant height (**a**), fresh weight (FW) (**b**), dry weight (DW) (**c**), and the levels of total chlorophyll (**d**) in leaves of rice seedlings exposed to 0, 50, 100 and 200 μM Ni for a period of 14 days. Bars represent means ± standard deviations (SDs) of three independent replications (*n* = 3). Means followed by the same letter did not significantly differ among the treatments at *p* < 0.05, according to Fisher’s least significant difference test.

To calculate the toxic effects of Ni on rice growth, we calculated the plant height, FWs, and DWs of the seedlings on day 14 of Ni application. The plant height was reduced by 13%, 26%, and 39% at 50, 100, and 200 μM Ni, respectively, in comparison with the control (Figure 2a). The incremental rise in Ni concentration in the solution seriously impaired the rice seedling biomass. Compared with the control, the reduction in FW was 12%, 43%, and 55%, and the reduction in DW was 21%, 40%, and 57% at 50, 100, and 200 μM Ni, respectively (Figure 2b,c). In the Ni-treated seedlings, the total Chl content decreased by 6%, 14%, and 19% at 50, 100, and 200 μM Ni, respectively, relative to the control (Figure 2d).

### 2.3. Effects of Ni Stress on NO_3_^−^ and NH_4_^+^ Concentrations

**Figure 3:**
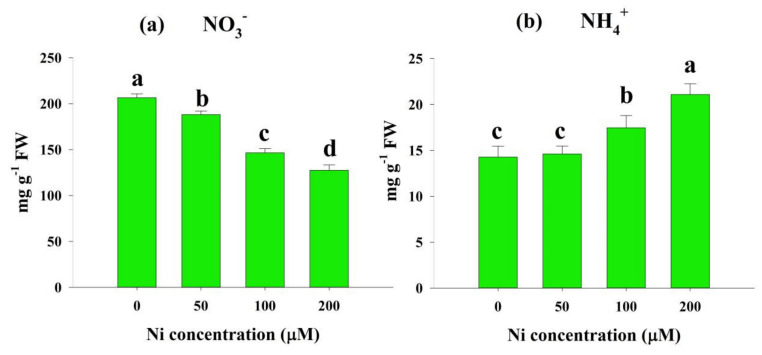
Effects of nickel (Ni) on the concentrations of nitrate (NO_3_^−^) (**a**) and ammonium (NH_4_^+^) (**b**) in the leaves of rice seedlings exposed to 0, 50, 100 and 200 μM Ni for a period of 14 days. Bars represent means ± standard deviations (SDs) of three independent replications (*n* = 3). Means followed by the same letter did not significantly differ among the treatments at *p* < 0.05, according to Fisher’s least significant difference test.

Ni-stressed seedlings showed a varied pattern in the concentrations of NO_3_^−^ and NH_4_^+^ in the rice leaves. The NO_3_^−^ concentration was decreased by 9%, 29%, and 38% at 50, 100, and 200 μM Ni, respectively, with respect to the control (Figure 3a). By contrast, the Ni levels raised the NH_4_^+^ concentration by 2%, 22%, and 48%, respectively, compared with the control (Figure 3b).

### 2.4. Effects of Ni Stress on Enzymes Involved in N Metabolism

**Figure 4:**
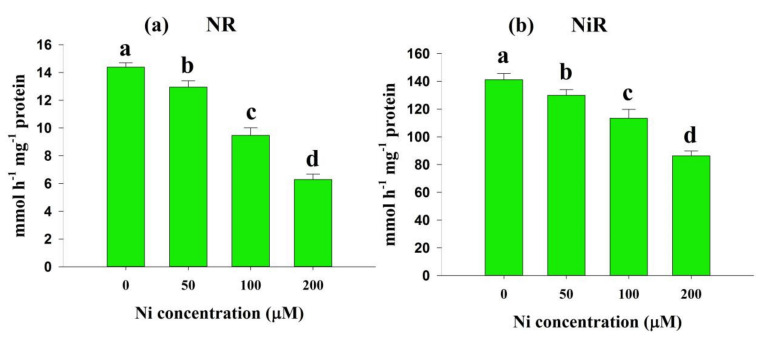
Effects of nickel (Ni) on the activity of nitrate reductase (NR) (**a**)**,** and nitrite reductase (NiR) (**b**) in the leaves of rice seedlings exposed to 0, 50, 100 and 200 μM Ni for a period of 14 days. Bars represent means ± standard deviations (SDs) of three independent replications (*n* = 3). Means followed by the same letter did not significantly differ among the treatments at *p* < 0.05, according to Fisher’s least significant difference test.

Ni-stressed rice seedlings showed declining levels of NR and NiR activities as the Ni concentration increased. The NR activity decreased by 10%, 34%, and 56%, and the NiR activity by 8%, 20%, and 39% at 50, 100, and 200 μM Ni, respectively, compared with the control (Figure 4a,b).

**Figure 5:**
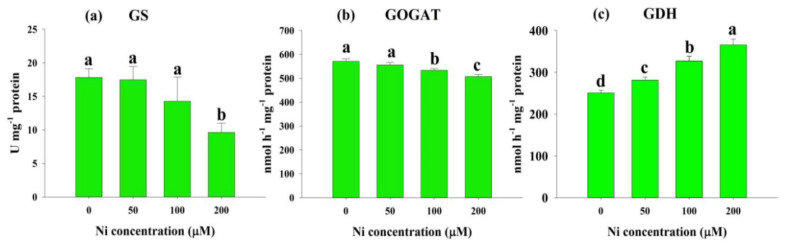
Effects of nickel (Ni) on the activity of glutamine synthetase (GS) (**a**), glutamate synthase (GOGAT) (**b**), and glutamate dehydrogenase (GDH) (**c**) in the leaves of rice seedlings exposed to 0, 50, 100 and 200 μM Ni for a period of 14 days. Bars represent means ± standard deviations (SDs) of three independent replications (*n* = 3). Means followed by the same letter did not significantly differ among the treatments at *p* < 0.05, according to Fisher’s least significant difference test.

Similarly to the NR and NiR activities, the GS activities and GOGAT activities were also inhibited upon a gradual increase in Ni levels by 2%, 17% and 44%, and 3%, 7% and 11% at 50, 100, and 200 μM Ni, respectively, in comparison with the control (Figure 5a,b). Ni stress resulted in a sharp rise in the activity of GDH, indicating a relatively linear association between the activity of GDH and the concentration of Ni in the culture medium [12%, 31% and 46% at 50 μM, 100 μM and 200 μM Ni, respectively] (Figure 5c).

**Figure 6:**
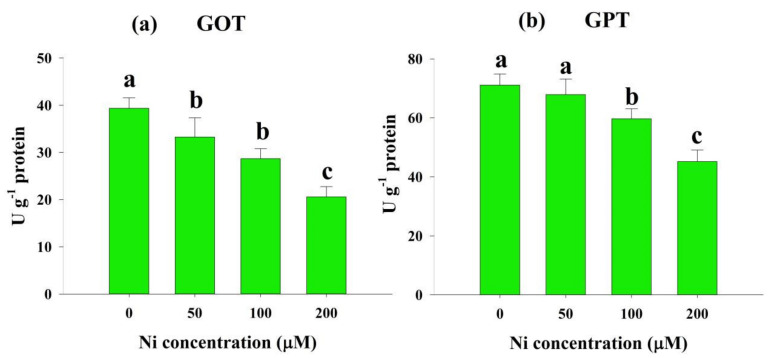
Effects of nickel (Ni) on the activity of glutamic oxaloacetic transaminase (GOT) (**a**) and glutamic pyruvic transaminase (GPT) (**b**) in the leaves of rice seedlings exposed to 0, 50, 100 and 200 μM Ni for a period of 14 days. Bars represent means ± standard deviations (SDs) of three independent replications (*n* = 3). Means followed by the same letter did not significantly differ among the treatments at *p* < 0.05, according to Fisher’s least significant difference test.

GOT and GPT activities, related to amino acid recycling, were significantly impaired by Ni stress. Compared with the control, Ni stress induced a significant reduction in GOT activity by 15%, 27%, and 48%, as well as in GPT activity by 5%, 16%, and 36%, respectively (Figure 6a,b).

## 3. Discussion

Plant growth and development was studied by varying Ni concentrations in a nutrient solution, with a gradient of Ni concentration. We observed that Ni stress causes phenotypic aberrations in rice, depending on the dose (Figure 1a). To support our findings, Ni-induced symptoms were also noted in various plant species, such as *O. sativa* L., *Glycine max* L., and *T. aestivum* L. [21,22]. The Ni concentration was increased in the shoot and root, according to Ni uptake (Figure 1b,c), and these results are consistent with those of *G. max* L. [22], *T. aestivum* L. [23], and rice [24]. Paddy rice is generally characterized by the formation of Fe plaque on the root surfaces. As a result of the continuous exchange of O_2_ from the rice shoots to the roots, Fe oxyhydroxides accumulate in the rhizosphere, which causes Fe plaques to develop around the roots [25]. Because the Fe plaque binds heavy metals, it minimizes their translocation into the shoots [26]. Thus, rice roots retain more Ni concentrations as compared to the shoot. Studies have demonstrated that Ni may contribute to plant growth and biomass in species such as *S. tuberosum* L., *T. aestivum* L., and *G. hirsutum* L., at low concentrations [11]. However, most plant species can exert toxicity if their accumulation is beyond the tolerable range [3]. The results of this study showed that the rice seedlings suffered growth retardation and reduced biomass with increasing concentrations of Ni in the solution (Figure 2a–c), as was shown by previous studies on *G. hirsutum* L. [27] and *L. esculentum* L. [28]. Consequently, the reduction in biomass from Ni-mediated stress could be due to the reduction in nutrient acquisition, essential for physiological functions in plants, or the oxidative stress triggered by Ni stress in plants [7,29].

The rate of photosynthesis affects plant growth and biomass by affecting the levels of carbohydrates and proteins. The content of total Chl in this study, as indicated in Figure 2d, decreased significantly following Ni administration. These findings confirm recent research showing that high Ni concentrations affect the total Chl content, limiting photosynthetic activity [8,22]. According to our previous studies, Ni stress decreased Chl a, Chl b and the total Chl content, and may also cause deformed stomata in the leaves, in addition to the chloroplast distortion observed, and may contribute to the decline in total chlorophyll levels [3,6,8,28]. Moreover, our study showed that under Ni stress (200 uM), gas exchange parameters, such as net photosynthetic rate (Pn), stomatal conductance (Gs), transpiration rate (Tr), and intercellular CO_2_ concentration (Ci), were decreased [8].

N metabolism is a fundamental physiological process in plants and is a crucial component of geochemical cycling [19]. In the majority of plants, the consumption or production of biochemically produced ammonium nitrate is linked to the concerted action of the NR/NiR cycle, composed of cytosolic NR and plastidial NiR, and to the subsequent action of GS and GOGAT, which comprise the GS/GOGAT cycle [19]. In this study, the NO_3_^−^ concentration in rice leaves was found to decrease significantly as the application of Ni in the solution increased (Figure 3a). In support of our findings, previous reports also demonstrated a decrease in NO_3_^−^ concentration in different plant species, such as *O. sativa* L. [30] and *T. aestivum* L. [23]. It is recommended that higher Ni concentrations limit the uptake and transport of NO_3_^−^ from roots. Similar to the NO_3_^−^ concentration, we observed a significant reduction in NR activity in the rice leaves (Figure 4a).

As a critical enzyme in N metabolism, NR catalyzes the reduction of NO_3_^−^ into NO_2_^−^ and is vulnerable to environmental stress conditions [31]. Our findings are consistent with earlier studies that found a reduction in NR activity in the presence of Ni stress (Figure 4a) [23,30]. It is suggested that a decline in NR activity could be caused by disrupting coordination among carbon, sulfur, and N metabolic pathways, along with the decline in NO_3_^−^ content [32]. Furthermore, this reduced NR activity may be attributed to one or more of the following: (1) a low affinity for NO_3_^−^ ions, (2) an increase in reactive oxygen species (ROS), and (3) reduced availability of NO_3_^−^ to plants during stressful conditions [33]. Additionally, Sharma and Dubey (2005) [33] demonstrated that the NR enzyme is susceptible to H_2_O_2_. Our previous studies observed a significant enhancement in endogenous accumulation of H_2_O_2_ in *O. sativa* L. under Ni stress conditions [3,8]. Therefore, the inhibition of NR activity may be associated with an increased reactive oxygen species content in rice leaves. In parallel to the decreased NR activity, we also observed a drastic decline in NiR activity with the increase in Ni concentration in the solution (Figure 4b), as supported by the previous study on *T. aestivum* L. [23]. NiR is an enzyme that catalyzes the reduction of NO_2_^−^ into NH_4_^+^. NR-catalyzed NO_3_^−^ reduction might cause a decline in NiR activity, primarily because of a reduced availability of NO_2_^−^ ions.

In the present study, Ni stress significantly increased the NH_4_^+^ content at all doses of Ni (Figure 3b), as was observed in other studies involving rice [30] and wheat [23]. Higher NH_4_^+^ accumulation within a cell is toxic, creating various damage, such as disturbance in osmotic balance, intracellular pH alteration, nutrient deficiency, ATP synthesis inhibition, and necrosis, ultimately restricting secondary growth [30]. This may be due to increased protease activity, hydrolysis of N-containing metabolites, and free amino acids [31]. The GS converts NH_4_^+^ to glutamine, which is then converted by GOGAT to glutamate. The GDH function is only activated when the GS/GOGAT cycle is blocked, and it is only activated under stressful circumstances [34]. We also detected inhibition in GS activity with augmented Ni doses in the present study (Figure 5a). Our findings follow those of Kevrešan et al. (1998) [35], who showed that Ni stress significantly reduced the GS activity of sugar beet leaves. Similar to GS activity, we observed that GOGAT activity also decreased significantly under Ni stress conditions (Figure 5b). Decreased GOGAT activity was also reported in different plant species when exposed to toxic levels of heavy metals [36,37]. It has been suggested that the decline in GOGAT activity is due to the decline in a process that initiates NO_3_^−^ uptake, and continues until the participation of NH_4_^+^ in the organic structure substances [36]. In contrast to GS and GOGAT activity, we detected a sharp increase in GDH activity with Ni application in the growth medium (Figure 5c). In accordance with our findings, many previous reports showed enhancement of GDH activity under various stress conditions in plants [38]. The ROS produced by abiotic stresses are believed to trigger glutamate synthesis by increasing GDH expression [39]. Furthermore, aminotransferases (GOT and GPT) play a critical role in glutamate metabolism, as they allow glutamate groups to be converted into other amino acids [40]. It has been found that GOT and GPT catalyze the conversion of glutamate to alanine and aspartate. Ni stress inhibited the activity of GOT and GPT in rice leaves in this study (Figure 6a,b). It has been suggested that Ni stress reduces GOT and GPT activities by impairing GS and GOGAT activities. After N is taken up by plants, glutamate is the first amino acid synthesized from it [38], and it is produced principally by NH_4_^+^ assimilation, catalyzed by these enzymes [41].

## 4. Materials and Methods

### 4.1. Plant Materials and Experimental Setup

Rice seeds (*Oryza sativa* L.), cultivar yangliangyou 6, were cleaned with H_2_O_2_ (10% (*v/v*)) for 10 min, followed by repeated washing and soaking in deionized water for 24 h. Afterward, seeds were placed on plastic nets placed on top of plastic pots containing deionized water and placed in darkness at 28 ± 2 °C. Twelve uniformly sized seedlings were moved after four days into a 4 L plastic box containing a 50% nutrient solution, and the pH was adjusted to 6.0 using either NaOH or HCl, as previously described [8]. Following 10 days of acclimatization, the nutrient solution was upgraded to 100%. The nutrient solution contained the following elements (in mg L^−1^): 40 N, 10 P, 40 K, 40 Ca, 40 Mg, 0.5 Mn, 0.05 Mo, 0.2 B, 0.01 Zn, 0.01 Cu, and 2 Fe, which were added in the form of NH_4_NO_3_, NaHPO_4_∙2H_2_O, K_2_SO_4_, CaCl_2_, MgSO_4_∙7H_2_O, MnCl_2_∙4H_2_O, (NH_4_)_6_Mo_7_O_4_∙4H_2_O, H_3_BO_3_, ZnSO_4_∙7H_2_O, CuSO_4_∙5H_2_O, FeCl_3_∙6H_2_O, and citric acid (monohydrate), respectively [20]. 

Plants were treated with Ni (NiSO_4_∙6H_2_O) at concentrations of 50, 100, and 200 μM, while rice seedlings without Ni concentration served as a control. The nutrient solutions were renewed every 3 days, and the seedlings were removed after the 14th day of Ni application. The trial was conducted according to a completely randomized design (CRD), with three replications. The experiment was carried out at Huazhong Agricultural University, Wuhan, China, and the seedlings were grown in a greenhouse at 28 ± 1 °C with a relative humidity of 80%, under a light intensity of 820 μmol m^−2^ s^−1^ (16/8 h day/night).

### 4.2. Determination of Plant Growth

After 14 days of treatment, the plant height and root length of the rice seedlings were measured using a ruler. After that, each seedling was dissected, washed with deionized water, then dried with tissue paper to remove excess surface water. To eliminate external Ni adsorbed at the root surface, roots were first washed with tap water, then with distilled water, and finally with 0.01 M HCl for approximately 5 s [42]. Following the determination of fresh weight, the roots and shoots of the rice seedlings were placed at 65 °C for 72 h so that their dry weight could be determined.

### 4.3. Determination of Total Chl Content

To determine the total content of Chl in the rice leaves, we measured the absorbance of 80% (*v/v*) acetone-extracted supernatant at 663, 645, and 470 nm, using the formulas suggested by Lichtenthaler and Wellburn (1983) [43].

### 4.4. Quantification of Ni Content

In order to determine the Ni content of the roots and shoots, they were oven-dried and then digested at 140 °C using a concentrated acid solution mixture (HNO_3_:HClO_4_ at 4:1). The resulting transparent liquids were used to determine the Ni concentration in the roots and shoots of the rice seedlings, using an atomic absorption spectrophotometer (AAS: Agilent Technologies, 200 series AA, Santa Clara, United States).

### 4.5. NO_3_^−^ and NH_4_^+^ Content Determination

The kits for NO_3_^−^ (ZXTD-2-G) and NH_4_^+^ (ZATD-2-G), offered by Comin Biotechnology Co., Ltd., Suzhou, China (http://www.cominbio.com) were used to evaluate the NO_3_^−^ and NH_4_^+^ quality of the fresh rice leaves. The instructions given by the manufacturer were strictly followed, and for NO_3_^−^ N and NH_4_^+^ N, respectively, the absorbance was read at 580 and 410 nm, and the units for both parameters were expressed in μg g^−1^ FW.

### 4.6. Measurement of N Metabolism-Related Enzyme Activities

Each frozen sample was ground in liquid nitrogen, and weighed within a range of 0.5 to 1 g. The enzymes were extracted in ice baths and then determined using the corresponding detection kit, according to the manufacturer’s instructions. The rice plants’ fresh leaves were used to measure the N metabolizing enzymes, such as NR, NiR, GS, GOGAT, GDH, GOT, and GPT; testing kits were purchased from Comin Biotechnology Co., Ltd., Suzhou, China (http://www.cominbio.com). To determine the activities of NR and NiR, the kits NR-2-Y and NIR-2-G were used, and the principle of measurement used was based on the concentration of enzymes needed to generate 1 μmol of NO_2_ per h per milligram of protein for both enzymes, which was quantified as one unit (U) of enzyme activity. Similarly, GS activity was tested using the GS-2-Y detection kit, and one U of enzyme activity was described in the per mL reaction system as per milligram protein required to adjust the absorption by 0.01 per min at 540 nm. The GOGAT enzyme level was investigated using the detection kit (GOGAT-2-Y), and the activity of one U enzyme was measured as 1 nmol NADH per minute per milligram of protein. The activity of GDH was monitored using the GDH-2-Y kit, and the principle of measurement was based on the enzyme level per milligram of tissue protein required to alter absorption by 0.01 per minute. The kits for GOT (GOT-2-Y) and GPT (GPT-2-Y) were used to investigate GOT and GPT enzyme levels. To make the absorption shift of 0.01 per minute, one U of enzyme activity was calculated per gram of protein in the reaction system.

### 4.7. Statistical Analysis

Treatment differences were analyzed for significance (*p* < 0.05) using a one-way analysis of variance (ANOVA) along with Fisher’s least significant test (LSD) performed with GraphPad Prism 8 software (GraphPad Software, Inc., La Jolla, CA, USA).

## 5. Conclusions

Collectively, higher levels of Ni have caused serious effects by altering several plant physiological and biochemical processes. Excess Ni exposure inhibited growth and reduced biomass, a phenomenon that was directly related to an increase in Ni concentration in the root and shoot tissue, as well as the depletion of photosynthetic pigments. Additionally, higher Ni concentrations influenced all the steps involved in N assimilation in rice leaves. As a result, NO_3_^−^ was reduced, and various enzyme activities, such as NR, NiR, GS, GOGAT, GOT, and GPT, were decreased, while the NH_4_^+^ concentration and GDH activity were increased. In summary, the findings suggest that Ni toxicity may cause desynchronization of the enzymes necessary for N assimilation in plants, subsequently having a negative impact on plant growth.

## Data Availability

The original contributions presented in the study are included in the article, further inquiries can be directed to the corresponding authors.
